# Effect of the Duration of Intraretinal or Subretinal Fluid on the Response to Treatment in Undertreated Age-Related Macular Degeneration

**DOI:** 10.1155/2020/5308597

**Published:** 2020-07-26

**Authors:** Izumi Yoshida, Masashi Sakamoto, Asao Sakai, Takatoshi Maeno

**Affiliations:** Sakura Medical Center, Toho University, Sakura-shi, Chiba, Japan

## Abstract

We investigated the association between the duration of intraretinal fluid (IRF) or subretinal fluid (SRF) and the response to antivascular endothelial growth factor injection in patients with undertreated age-related macular degeneration (ARMD). The Ethics Committee of Toho University Sakura Medical Center approved this study (no. S18030). Eighty eyes of ARMD patients with VA ≤20/100 were retrospectively assessed. Each injection's efficacy was classified, and the fluid accumulation prior to each injection was evaluated. The effect changes following to accumulated IRF, SRF, the longest persistent IRF period (≥10 months), and their determining factors were evaluated. Throughout observation, acquired refractoriness was rarely associated with increased accumulation of IRF or SRF. The injection span had a tendency to be short, and the polypoidal choroidal vasculopathy and occult choroidal neovasculopathy (CNV) proportions had a tendency to be higher among patients with diminished effects than among those with maintained effects. VA differed significantly with continuous IRF duration, but not with accumulated fluid. The diminishing effect of injections during long-standing IRF was rarely associated with undertreatment. The mechanism underlying acquired refractoriness remains unknown; the effect change demonstrated various patterns, including diminished and improved responses. The longest continuous IRF duration was associated with VA decline. Shortening the duration of continuous IRF may be necessary.

## 1. Introduction

The treat-and-extend method for age-related macular degeneration (ARMD) is highly versatile and widely used [1, 2]. However, there are few reports regarding treatment effects in patients pre-treated with pro re nata or less treatment over a long period [3, 4] with pre-existing poor visual acuity (VA). The SEVEN-UP Study reported that, after about 7.3 years, 37% of 65 eyes had best-corrected visual acuity (BCVA) of 20/200 or worse as a result of spontaneous treatment by individual physicians after completion of the HORIZON study. This indicated that 10 patients with poor vision (mean: 21.1 letters) were never recommended for treatment during a period of 3.5 years [5].

Some reports have suggested stopping treatment for very advanced cases of ARMD [5, 6]. However, we have encountered massive intraretinal fluid (IRF) after long periods without treatment. We considered that the duration of continuous fluid would reduce the efficacy of injections caused by destroyed retinal structures. Additionally, in long-standing ARMD, wherein fluid appearance and resolution repeated, would refractoriness be influenced by the accumulation of these appearance-to-disappearance periods? Moreover, would refractoriness be influenced by the absence of continuous injection, whereas each injection could not resolve fluids? Does previous photodynamic therapy (PDT) have any influence? Additionally, if there was any absolute length of continuous IRF or accumulation of fluids, against fluids after these lengths, injection would become invalid caused by retinal destruction, we should not administer invalid injection after the limit, and on the other hand, we should administer frequent injection before the limit. To our knowledge, no previous reports have investigated these issues. Moreover, as we have observed sudden improvement in refractory fluid after an extended period, we questioned whether there are rules or patterns regarding the changing effect of antivascular endothelial growth factor (anti-VEGF) injections. If present, injection protocols should conform to the discovered rule, regardless of the length of the refractory period.

The purpose of this study was to investigate the diminishing effect of treatment on accumulated or long-continuous fluid; the rule or pattern of change in injection efficacy, if present; and the influence of previous injection span or PDT on the efficacy of injections in patients with ARMD. Additionally, we investigated the relationship between continuous IRF versus repeated accumulation of IRF or SRF and VA change.

## 2. Materials and Methods

The Ethics Committee of Toho University Sakura Medical Center approved this study (no. S18030). The procedures used conformed to the tenets of the Declaration of Helsinki. The study design was explained on the Sakura Medical Center website in accordance with the guidelines for clinical research set out by the Japanese Ministry of Health, Labour and Welfare. All patients provided written informed consent for treatments; all private patient information was excluded from the database. The use of anonymous information was approved by the IRB without the need to seek further consent.

We examined consecutive patients with ARMD whose most recent visit to our hospital was from February 2018 to February 2019. As we aimed to investigate undertreated patients with poor VA and the majority of previous studies defined baseline VA as better than 20/100 [1, 5, 7, 8] and a few reports suggested there were benefits in treating eyes with VA of ≤20/200 [2, 9], we investigated patients with VA ≤20/100. We excluded patients with an observation period of less than 10 months, who missed follow-up visits during the observation period, or who underwent direct laser photocoagulation for choroidal neovasculopathy (CNV), gas injection, and vitrectomy.

**Figure 1:**
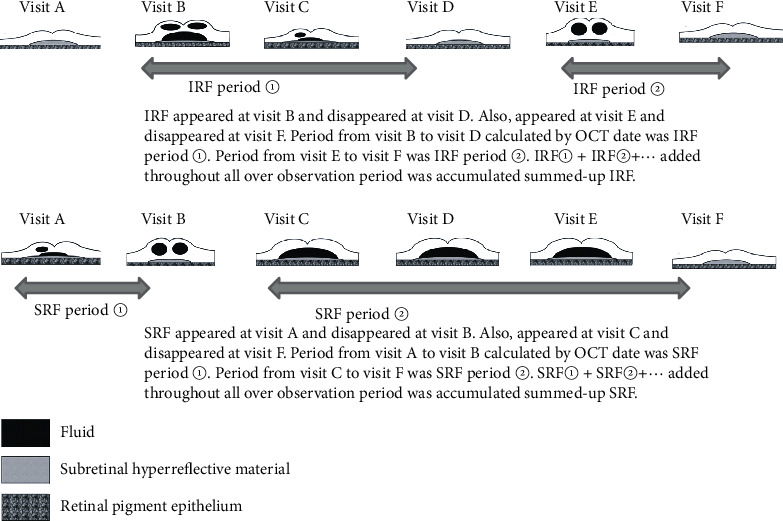
Accumulated summed up IRF or SRF.

**Figure 2:**
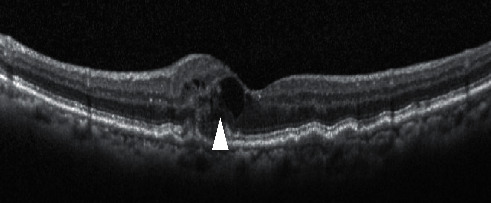
White arrow showing intraretinal fluid (IRF).

**Figure 3:**
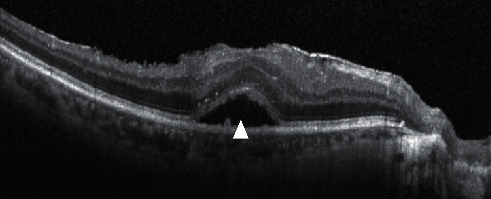
White arrow showing subretinal fluid (SRF).

**Figure 4:**
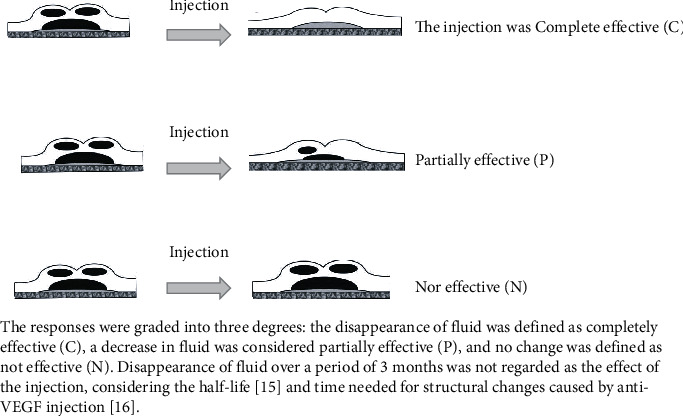
Grading of response for injection.

We retrospectively examined each patient's whole treatment duration from their medical records. We calculated durations from fluid appearance until disappearance for the overall observation period (from the first invitation to the last invitation) (Figure 1) of intraretinal fluid (IRF) (Figure 2) and subretinal fluid (SRF) (Figure 3) and defined the response of each injection as one of the three degrees (Figure 4).

The responses were classified into three degrees: the disappearance of fluid was completely effective (C), the decrease in fluid was considered partially effective (P), and no change was defined as not effective (N). The disappearance of fluid over a period of 3 months was not regarded as the effect of the injection, considering its half-life [10] and the time needed for structural changes caused by antivascular endothelial growth factor (anti-VEGF) injection [11].

For the first, we determined if there was a relationship between the response and the overall summed up duration of fluids up to the time point of injection.

**Figure 5:**
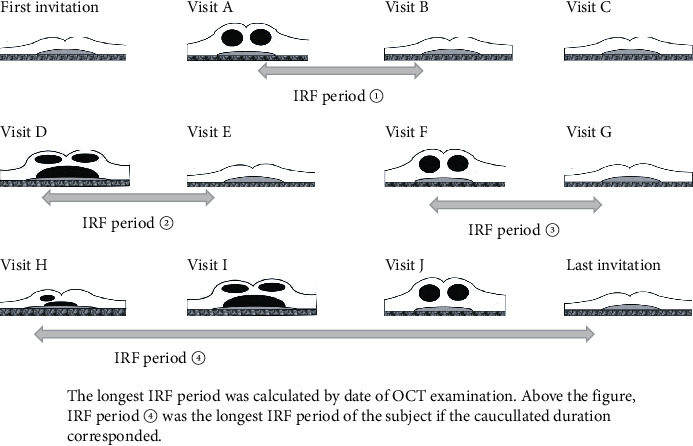
The longest IRF period.

Second, we examined the occurrence of diminishing response during the period of long-continuous fluid. The longest IRF period was calculated by the date of optical coherence tomography (OCT) for each patient (Figure 5). Long continuous SRF was rare in long-standing ARMD; thus, we examined only continuous IRF. In various studies, subjects with exudation present over 6–12 months were considered as having long-standing fluid, which could influence visual outcomes [14–21]; we investigated continuous IRF ≥10 months. During each patient's longest continuous IRF, the occurrence of diminishing response (C ⟶ P, P ⟶ N, or C ⟶ N) was examined. Moreover, the number of injections and their span during the fluid period, the previous summed up repeated IRF and SRF duration, PDT performed previously or during the period, and the type of CNV were examined as determining factors.

Third, we examined the occurrence of response change during the whole period and evaluated the regularity of the time point and any determining factors such as number or span of injections, summed up repeated IRF or SRF, and previous PDT about C ⟶ P, P ⟶ C, C ⟶ P ⟶ C, and P ⟶ C ⟶ P pattern.

At last, we examined the relationships between changes in the logarithm of the minimum angle of resolution (logMAR) of the best-corrected visual acuity (BCVA) and the total accumulated IRF and SRF and the longest persistent IRF period over 36 months from the time that the BCVA of each eye first declined to ≤20/100. Eyes with shorter observation periods and eyes that had undergone cataract surgery during the 36-month period were not included in this analysis.

We recorded the type of CNV, the presence or absence of persistent subretinal hyperreflective material (SHRM), total number of injections, span of injections (months/injection), number of PDT sessions, baseline VA, and VA at the last visit. Persistent SHRM [12] was defined as that still present at the last visit. The number of injections was the total number of both aflibercept and ranibizumab injections. Each duration was calculated based on optical coherence tomography (OCT), using the RTVue-100 (Optovue, Inc., Fremont, CA) and the Heidelberg Spectralis HRA + OCT (Heidelberg Engineering GmbH, Heidelberg, Germany).

### 2.1. Statistical Analysis

We applied the Mann–Whitney U test, Wilcoxon's signed-rank test, Fisher's exact test, the Kruskal–Wallis test, Cramer's V test, and Spearman's correlation coefficient by rank test using Statcel 3 software^®^ 2011 (OMS Publishing Inc., Saitama, Japan). *P* < 0.05 was considered to indicate significant differences.

## 3. Results

Table 1 shows the characteristics of the enrolled eyes. A total of eighty eyes of 72 patients were analysed. The mean injection span was 9.28 ± 18.44 months. The PCV and occult CNV were detected in 56/80 eyes (70%). Persistent SHRM was observed in 74/80 eyes (93%).

### 3.1. Accumulation of Repeated IRF or SRF Periods and Effect of Injections

Table 2 shows the accumulation of added IRF and effect of injections.

Table 3 shows the accumulation of added SRF and effect of injections.

The changes in the proportion of C, P, and N responses according to the total accumulated IRF and SRF up to 70 months seemed to have no accurate tendency for diminishing or increasing anti-VEGF response.

### 3.2. Occurrence of Diminishing Effect during the Longest IRF Period and Determining Factors

Thirty-seven eyes had the longest IRF periods of more than 10 months.

No C response was observed in four eyes after 70–90 months of continuous IRF.

Table 4 shows the differences between the maintained and diminished groups among the longest IRF.

Nineteen eyes were included in the maintained group, and nine eyes were included in the diminished group. Four eyes that were injection naïve and five eyes that had received a single injection were not included in the analyses. The injection span had a tendency to be shorter in the diminished group than in the maintained group, but difference was not significant (*P*=0.192). The total accumulation of IRF period was much shorter in the diminished group than in the maintained group (*P*=0.018). PDT during the longest IRF period was performed in 2/9 (18.2%) eyes in the diminished group and never in the maintained group (*P*=0.178). Polypoidal choroidal vasculopathy (PCV) or occult AMD were present in 8/9 (89%) eyes in the diminished group, and the proportion had a tendency to be higher than 10/19 (53%) eyes in the maintained group, but difference was not significant (*P*=0.147).

### 3.3. Changing of Injection Effects during Whole Observation Periods and Any Patterns

A C response was maintained in 15 eyes throughout the study period. Efficacy changed from C to P, and thereafter did not change, in four eyes. In seven eyes, the following was observed: C ⟶ P ⟶ C. In 17 eyes, the following was observed: P ⟶ C. In three eyes, the following was observed: P ⟶ C ⟶ P. In 11 eyes, a P response was observed throughout the study. Other more complex patterns were seen in 15 eyes, and 8 eyes received ≤3 injections.

Table 5 shows the timing of each pattern change from the first visit, total accumulated IRF up to the change, total accumulated SRF up to the change, number of injections, injection span, aflibercept/ranibizumab use, switching drugs, and previous PDT.

### 3.4. VA Change and Accumulated IRF, SRF, and Continuous IRF

Table 6 shows the correlations between logMAR BCVA changes at 36 months from the point where the BCVA of each eye declined to ≤20/100.

BCVA change had no significant correlation with accumulated IRF or SRF but was somewhat correlated with the longest persistent IRF (*r*_s_ = 0.1091, 0.1384, and 0.2979; *P*=0.440, 0.328,  and 0.035, respectively).

## 4. Discussion

Algvere et al. reported that, in 13 subjects, VA with ARMD lasting 13–30 months was improved after 6 months of treatment [13]. Gianniou et al. reported that, among 76 subjects with persistent fluid after 12-month visits with monthly injections, VA improved during the subsequent 36 months [14]. Takahashi et al. reported that the mean total injection delay per year in a rural hospital was 147 days, although VA improved [15]. These reports suggest the effects of treatment for long-standing ARMD and relationships between duration of IRF or SRF and VA, although the durations of fluids were not calculated. There have been no previous reports about relationships between the effect of injection and accumulated fluid calculated by date on OCT.

In the present study, mean injection span was found to be much longer than that of other reports [1–4]. It is evident that intraretinal and/or subretinal fluid was caused by fewer intraretinal anti-VEGF injections than actually needed, and we would need to investigate the efficacy of further treatment in these subjects. Contrary to our hypothesis, we found that long-standing oedema or lesser injections did not seem to correlate with refractoriness to further anti-VEGF injection. For the first, there seemed to be no accurate diminishing effect according to summed up IRF and SRF accumulated up to 70 months. Second, among eyes with longest IRF ≥10 months, the efficacy was retained for 19/28 eyes, and total added SRF seemed to be poorly associated with the diminished effect, whereas 4 eyes with after 70–90 months of continuous IRF had no C response. Moreover, we found that the injection span had a tendency to be shorter, and the proportion of occult CNV and PCV had a tendency to be higher in the diminished group than in the maintained group, though these were not statistically significant. The proportion of PCV and occult CNV in whole subjects was also much higher than the usual proportions in the Japanese population [16]. Previous reports had stated that some subjects with PCV had refractory for anti-VEGF injection [17], and subjects with pigment epithelial detachment had worse VA prognosis [18]. Given that we investigated worse VA subjects, similar subjects with PCV or occult CNV would be included. Persistent SHRM was observed much more frequently in subjects of the present study than usual proportion [12] and would be induced by prolong exudate, though SHRM was reported to be observed more frequently in classic CNV in early periods [19]. Suzuki et al. reported that eyes with multiple polyps exhibited a higher prevalence of residual fluid after ranibizumab injection and a shorter mean interval between injections [7]. It is possible that similar subjects were included in the diminished group in our study. Interestingly, accumulated IRF was much shorter in the diminished group, whereas SRF was not. Perhaps, periods of increasing refractoriness were mainly in the earlier periods of appearance of IRF, after SRF had continued to some extent.

Additionally, previous PDT seems to be rarely associated with maintaining the effect of anti-VEGF injections. Though the EVEREST study [20] reported that ranibizumab and PDT combination therapy was superior to monotherapy, these efficacies could be accurate with more constant injection than our subjects received.

In terms of effect change patterns, the timing of a C ⟶ P change had a tendency to occur later than a P ⟶ C change, though this difference was not statistically significant. It is possible that a P ⟶ C change was caused after several injections in individuals with excessive inflammatory cytokines [21], while a C ⟶ P change was caused by tachyphylaxis when the neutralizing antibody was produced [22]. Additionally, the injection span up to P ⟶ C in a C ⟶ P ⟶ C pattern had a tendency to be shorter than that up to C ⟶ P in a P ⟶ C ⟶ P pattern, but this was not statistically significant. It is possible that, in these later changes, the initial cytokines or neutralizing antibody did not change significantly, and the injection span directly influenced the effect. The P ⟶ C change in a P ⟶ C ⟶ P pattern commonly occurred in conjunction with a high aflibercept/ranibizumab ratio than with a C ⟶ P change in a C ⟶ P ⟶ C pattern. These different effects according to pharmacological differences would occur in the earlier periods.

In this study, there was a little relationship between VA changes during the 36-month period in those subjects with a baseline VA ≤ 20/100 and the total accumulated IRF or SRF. Nevertheless, a relationship between minimized exudative duration and better VA has been frequently reported [1–5]. In our study, the primary reason for the weak association between repeated exudates and VA decline is that comparisons were performed among undertreated individuals with worse VA than that reported in previous studies [1, 5, 7, 8]. Currently, it is well known that the presence of IRF requires immediate treatment, and in contrast, SRF would sometimes be tolerated [19, 23]. Although, in the present study, neither accumulated IRF or accumulated SRF accurately caused significant VA decreasing or refractory to injection. It could be conjectured that changes in retinal structure would be slowly exacerbated by repeated accumulation of IRF and SRF; however, the rough basal structures and functions would be retained to some extent in those patients. On the other hand, there was also a significant correlation between the logMAR change and the longest persistent IRF duration. This emphasizes the need to avoid long persistent IRF. Continuous IRF could damage such basal structures and lead to further destruction and an irreversible condition.

This study highlighted some points about managing subjects who already have poor VA and who have been previously undertreated. First, the use of anti-VEGF injections should not be dismissed based on previous accumulation of SRF or IRF and fewer injection. Second, refractoriness could be caused by various factors, and the exact mechanism remains unknown. The effect of injection could change during the follow-up, including both diminishing and improving patterns, although there did not appear any unalterable patterns based on the previous courses that followed injection. Third, in patients with long-standing persistent IRF, refractoriness may be caused by high primary CNV activity, particularly in cases with occult CNV or PCV. Fourth, efforts should be made to shorten the duration of continuous IRF. Perhaps, for long-standing subjects undertreated previously such as treat-and-extend was not necessary and intensive alterations of fluids once in several months was alternative. Further prospective studies would be needed to accurately reveal the relationship between fluids, injection refractoriness, and VA decline.

Our study had some limitations. First, accumulation of IRF or SRF was not examined over 70 months. A longer duration might cause refractoriness. Moreover, in four eyes after 70–90 months of continuous IRF, no C response was observed. Second, the variety of observation periods could have influenced the results. Third, this was a retrospective study, and the IRF or SRF periods were influenced by each patient's visit interval.

## 5. Conclusion

In conclusion, the diminished response to anti-VEGF treatments did not seem to correlate with accumulated fluid or few injections and may rather be associated with high CNV activity. The mechanism of acquired refractoriness to anti-VEGF remains unknown; moreover, the changes in effects demonstrated various patterns. The duration of continuous IRF was associated with VA decline. Our study findings indicate that the use of anti-VEGF injection should not be dismissed in cases with previous long-standing fluid. Additionally, it is necessary to endeavor to shorten the duration of continuous IRF. Furthermore, it should be kept in mind that both refractoriness and improved efficacy of the injection may be acquired.

## Figures and Tables

**Table 1:** Characteristics of subjects.

Eyes, *N*	80				
Sex	Males: 57		Females: 15		
Age	80.89 ± 6.49				
Type	Classic: 6				
	Occult: 20				
	PCV: 36				
	RAP: 4				
	Cannot be classified or none fluorescein angiography: 14				
Persistent SHRM (eyes)	74/80 (93%)				
Photodynamic therapy (eyes)	Never: 59	Once: 14		Twice: 5	Three times: 2
Total number of injections^*∗*^ (aflibercept or ranibizmab) (times)	Mean: 11.94 ± 6.43 (range, 0–28)				
Total observation period (months)	Mean: 65.63 ± 29.77 (range, 10–144 months)				
Injection span (months)^*∗*^	Mean: 9.28 ± 18.44 (range, 1.9–142)				
Baseline logMAR VA	0.564 ± 0.456				
Final logMAR VA	1.031 ± 0.381				

^*∗*^Two eyes never had gone for injection.

**Table 2:** Accumulation of summed up IRF and effect of injections.

	First injection	Summed up IRF 10 months	20 months	30 months	40 months	50 months	60 months	70 months
Complete effective (eyes)	39	25	18	10	3	2	2	3
Partially effective (eyes)	29	22	13	8	8	7	2	0
No effective (eyes)	3	3	1	0	0	0	2	1
All (eyes)	71	50	32	18	11	9	6	4

Note: the table shows the changes of the effect of injection on all subjects according to increasing summed up of all IRF duration. If no injection was performed at the time point, the subject was not counted at the vertical column.

**Table 3:** Accumulation of summed up SRF and effect of injections.

	First injection	Summed up SRF 10 months	20 months	30 months	40 months	50 months	60 months	70 months
Complete effective (eyes)	40	18	23	14	13	3	2	3
Partially effective (eyes)	29	31	22	13	7	10	3	1
No effective (eyes)	3	3	1	1	1	3	2	1
All (eyes)	72	52	46	28	21	16	7	5

Note: the table shows the changes of the effect of injection on all subjects according to increasing summed up of all SRF duration. If no injection was performed at the time point, the subject was not counted at the vertical column.

**Table 4:** Differences between diminished or maintained effect among IRF continuous over 10 months group.

	Effect maintained	Effect diminished	*P* value
Number (eye)	19/28 (67.9%)	9/28 (32.1%)	
Longest IRF (months)	26.78 ± 20.96 (range, 10–70)	53.56 ± 29.05 (range, 18–95)	0.005^※^
Injection number during longest IRF	3.68 ± 1.38 (range, 2–7)	11.33 ± 7.50 (range, 7–27)	＜0.0001^※^
Injection span (months)	7.59 ± 7.18 (range, 3.3–35)	6.55 ± 6.71 (range, 2.4–23)	0.192^※^
PDT previous longest IRF (eyes)	4/19 (21.1%)	2/9 (22.2%)	0.673^*∗*^
PDT during longest IRF (eyes)	0/19 (0%)	2/9 (18.2%)	0.178^*∗*^
Total added IRF previous longest IRF (months)	7.05 ± 9.48 (range, 0–37.5)	0.5 ± 0.90 (range, 0–2.5)	0.018^※^
Total added SRF previous longest IRF (months)	9.20 ± 9.52 (range, 0–29.5)	7.44 ± 10.58 (range, 0–28.5)	0.322^※^
Type of CNV (occult or PCV/others)	10/19 (53%) (PCV 8; occult 2)	8/9 (89%) (PCV 4; occult 4)	0.147^※^

Nine eyes with no injection or one injection were not included. Total added SRF was 0 if the longest IRF was the first IRF. ※Mann–Whitney U test. ^*∗*^Fisher's exact test.

**Table 5:** Characteristics of changing timing of each patterns of change.

	C ⟶ P	C ⟶ P in C ⟶ P ⟶ C	P ⟶ C in C ⟶ P ⟶ C	P ⟶ C	P ⟶ C in P ⟶ C ⟶ P	C ⟶ P in P ⟶ C ⟶ P
Number (eyes)	4	7	7	17	3	3
Timing of change from the first visit (months)	44.63 ± 20.12 (range, 25–44)^*∗*^	27.35 ± 21.39 (range, 4–67)	42.85 ± 17.96 (range, 23–77)	24.41 ± 42.03 (range, 3–48)^*∗*^	27.17 ± 10.21 (range, 15–39)	58.67 ± 22.11 (range, 28–77)
Accumulated IRF until the change (months)	9.0 ± 10.98 (range, 0–24)	11.04 ± 5.21 (range, 2–19)	21.46 ± 11.38 (range, 3–43)	10.04 ± 18.59 (range, 0–24)	8.17 ± 7.55 (range, 0–19)	19.33 ± 17.06 (range, 0–42)
Accumulated SRF until the change (months)	13.0 ± 6.98 (range, 5–22)	13.07 ± 11.22 (range, 2–30)	20.64 ± 12.44 (range, 2–40)	16.55 ± 11.26 (range, 5–41)	15.33 ± 6.38 (range, 7–23)	26.50 ± 16.68 (range, 5–50)
Number of injections until the change (times)	8.25 ± 4.57 (range, 3–14)	7.43 ± 4.44 (range, 3–14)	10.14 ± 4.36 (range, 5–17)	6.71 ± 3.60 (range, 3–18)	6.0 ± 2.45 (range, 3–9)	9.33 ± 3.40 (range, 6–14)
Injection span (months/number)	7.16 ± 4.98 (range, 3–12)	3.62 ± 1.43 (range, 1–5)	5.63 ± 2.81 (range, 3–11)^※^	3.76 ± 2.69 (range, 1–9)	6.12 ± 4.92 (range, 2–13)	9.24 ± 2.39 (range, 6–12)^※^
Aflibercrpt/ranibizmab	2/2	2/5^✳^	4/3	15/2	3/0^✳^	2/1
Switching ranibizumab ⟶ Aflibercept	1	1	2	3	0	0
Switching aflibercept ⟶ Ranibizmab	0	0	0	1	0	1
No switching at the point	3	6	5	13	3	2
Previous PDT	1/4	1/7	0/7	3/17	2/3	0/3

Vertical row shows the each pattern of change and horizontal line show the characteristics of the change timing. ^*∗*^*P*=0.060, ^※^*P*=0.087 (Mann–Whitney U-test), and ^✳^*P*=0.038 (Fisher's exact test). Other pairs were not significantly different (Mann–Whitney U test, Wilcoxon signed-rank test, Kruskal–Wallis test, and Cramer's V test). *Note*. C ⟶ P in C ⟶ P ⟶ C: whole change pattern is completely effective ⟶ partially effective ⟶ completely effective, and this row shows about effect change completely effective ⟶ partially effective.

**Table 6:** Visual acuity change from baseline 20/100 during the 36 months interval, the total accumulated IRF and SRF periods, and longest continuous IRF period.

VA change (*n* = 51) (logMAR BCVA change)	Parameter	Duration (months)	Rs	*P* value
0.0753 ± 0.384 (range, 1–1.18)	Total added IRF	17.73 ± 12.90 (0–36)	0.1091	0.440
	Total added SRF	15.96 ± 10.49 (0–36)	0.1384	0.328
	Longest continuous IRF	24.61 ± 29.29 (0–121.5)	0.2979	0.035

Spearman's correlation coefficient by rank test. BCVA, best-corrected visual acuity; IRF, intraretinal fluid; SRF, subretinal fluid. Eyes with shorter than 36 months of observation periods and eyes that had gone undergone cataract surgery during the 36-month period were not included in the analysis.

## Data Availability

The datasets generated and analysed during the current study are not publicly available because we are not able to permit any possibility of identifying persons from treatment history regardless of data anonymity, but data are available from the corresponding author upon reasonable request.
